# Deep learning visual field global index prediction with optical coherence tomography parameters in glaucoma patients

**DOI:** 10.1038/s41598-023-43104-y

**Published:** 2023-10-25

**Authors:** Dongbock Kim, Sat Byul Seo, Seong Joon Park, Hyun-kyung Cho

**Affiliations:** 1 Department of Mathematics Education, School of Education, Kyungnam University, 7 Kyugnamdaehak‑ro, Masanhappo‑gu, Changwon, Gyeongsangnam-do 51767 Republic of Korea; 2 Department of Ophthalmology, Gyeongsang National University Changwon Hospital, School of Medicine, Gyeongsang National University, 11 Samjeongja-ro, Seongsan-gu, Changwon, Gyeongsangnam-do 51472 Republic of Korea; 3 Institute of Health Sciences, School of Medicine, Gyeongsang National University, Jinju, Republic of Korea

**Keywords:** Optic nerve diseases, Machine learning

## Abstract

The aim of this study was to predict three visual filed (VF) global indexes, mean deviation (MD), pattern standard deviation (PSD), and visual field index (VFI), from optical coherence tomography (OCT) parameters including Bruch's Membrane Opening-Minimum Rim Width (BMO-MRW) and retinal nerve fiber layer (RNFL) based on a deep-learning model. Subjects consisted of 224 eyes with Glaucoma suspects (GS), 245 eyes with early NTG, 58 eyes with moderate stage of NTG, 36 eyes with PACG, 57 eyes with PEXG, and 99 eyes with POAG. A deep neural network (DNN) algorithm was developed to predict values of VF global indexes such as MD, VFI, and PSD. To evaluate performance of the model, mean absolute error (MAE) was determined. The MAE range of the DNN model on cross validation was 1.9–2.9 (dB) for MD, 1.6–2.0 (dB) for PSD, and 5.0 to 7.0 (%) for VFI. Ranges of Pearson’s correlation coefficients were 0.76–0.85, 0.74–0.82, and 0.70–0.81 for MD, PSD, and VFI, respectively. Our deep-learning model might be useful in the management of glaucoma for diagnosis and follow-up, especially in situations when immediate VF results are not available because VF test requires time and space with a subjective nature.

## Introduction

Glaucoma is caused by injuries to retinal ganglion cells (RGC) and their axons, leading to retinal nerve fiber layer (RNFL) deficit and neuroretinal rim (NRR) thinning that can result in visual field (VF) defects^1^. Measurement of peripapillary RNFL using optical coherence tomography (OCT) scan is a broadly accepted method for the quantitative assessment of structural damage in glaucoma^2^. Standard automated perimetry (SAP) is the standard method to detect and monitor functional VF defect in the management of glaucoma^3,4^. However, there are some intrinsic limitations of a VF test. First of all, this test has a subjective nature. Moreover, it has a high intra-subject variability (high test-to-test variability), a lengthy test time, and a necessity for a designated place to perform SAP^5,6^. Structure–function relationship is important in the understanding and management of glaucoma^7–10^. Detectable structural changes usually precede VF functional loss at each individual degree^10–14^.

**Figure 1:**
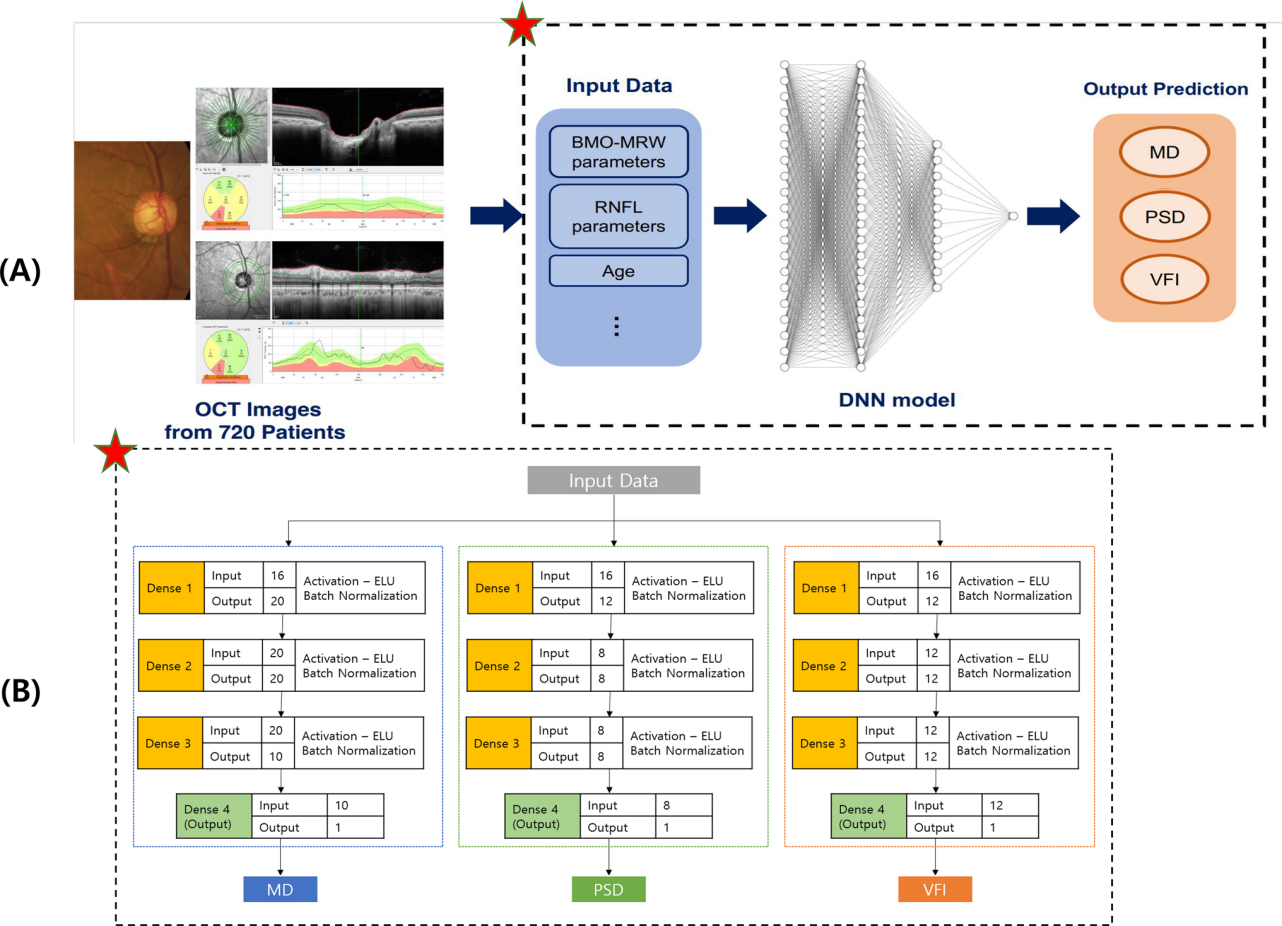
(**A**) Workflow of this study. Input data extracted from OCT images are used to predict VF indexes (MD, PSD, VFI) through a DNN model. Detailed structure of dashed line box with a red star is described in (**B**). (**B**) Detailed structure of the DNN model. Each number above each box represents the number of nodes in the prior layer. The number below each box means the number of nodes in the present layer. OCT = optical coherence tomography; MD = mean deviation; PSD = pattern standard deviation; VFI = visual field index; DNN = deep neural network.

Recently, spectral-domain OCT provides Bruch’s membrane opening-minimum rim width (BMO-MRW) as a new parameter in addition to conventional peripapillary RNFL. BMO-MRW measures the shortest length from the inner opening of BMO to the internal limiting membrane (Fig. 1A), which has been introduced for assessing optic nerve head^15–19^. BMO-MRW provides more accurate evaluation of the NRR than conventional optic disc inspection^15–20^. Previous studies have demonstrated that BMO-MRW showed superior diagnostic ability in glaucoma to previously used parameters of NRR^21–23^. BMO-MRW has also been reported to show a better structure–function relationship than other NRR parameters using conventional confocal scanning laser ophthalmoscopy or peripapillary RNFL^23,24^.

We have previously reported a high diagnostic performance in distinguishing early normal-tension glaucoma (NTG) from glaucoma suspect (GS) (AUC, 0.966) based on a deep learning model using OCT parameters of BMO-MRW, peripapillary RNFL, and color classification of RNFL^25^. Interestingly, BMO-MRW, as a single parameter, provided a higher diagnostic performance (AUC: 0.959) than RNFL alone (AUC: 0.914) and RNFL with its color code classification (AUC: 0.934)^25^. Moreover, BMO-MRW alone showed similar diagnostic performance to that of all three OCT parameters combined. These results suggest that BMO-based optic disc assessment might be a better evaluation for different aspects of the optic disc than conventional disc assessments in the diagnosis of glaucoma.

Previous structure–function studies have used deep learning models to predict global VF indexes including mean deviation (MD) from OCT-derived images such as RNFL thickness maps^26,27^. Other previous studies have predicted pointwise threshold of VF from OCT-derived image scans like peripapillary RNFL or macular ganglion cell complex thickness maps^28–31^. However, none of these previous studies included any information regarding BMO-MRW. Moreover, none predicted all three VF global indexes of MD, pattern standard deviation (PSD), and visual field index (VFI) from OCT-derived images or maps. Each global index of VF test has its own advantage, and therefore, only one index cannot tell all the aspects of VF test results. Actual figures of global indexes of VF could provide an outline of VF summary, which might be clinically useful in the management of glaucoma including diagnosis and detection of progression.

Thus, the aim of the present retrospective cross-sectional study was to predict three VF global indexes using deep-learning model from OCT-derived parameters of BMO-MRW and RNFL. We intended to assess the usefulness of this deep-learning model as a reference in glaucoma clinic. It might be beneficial in situations when immediate VF results are not available since VF test takes time and cooperation of the patient. We applied a deep-learning model to integrate all data available from spectral-domain OCT images to predict VF global indexes, which might be challenging for general physicians.

## Results

### Baseline characteristics of subjects

A total of 720 eyes (720 patients) with glaucoma and glaucoma suspect (GS) were included in the final analysis. Glaucoma diagnosis included early normal-tension glaucoma (NTG), moderate stage of NTG, pseudo exfoliation glaucoma (PEXG), primary angle closure glaucoma (PACG), and primary open angle glaucoma (POAG). The mean age of glaucoma patients was 53.7 ± 13.3 (mean ± standard deviation) years. Females accounted for 46% (328/720). Of all patients, 8.3% (60/720) had a family history of glaucoma. Baseline spherical equivalent (SE) was − 1.8 ± 2.9 diopters. Baseline intraocular pressure (IOP) was 15.6 ± 4.1 mmHg with central corneal thickness (CCT) of 542.0 ± 42.7 um. Baseline MD was − 4.5 ± 5.8 dB, PSD was 5.3 ± 4.2 dB, and VFI was 88.6 ± 17.0 dB. Baseline characteristics including VF global indexes for the training set and test set, respectively, are summarized in Table 1. Baseline OCT parameters of BMO-MRW and RNFL are demonstrated in Table 2.

**Table 1:** Baseline characteristics of included glaucoma patients.

Characteristics		Training set	Test set
Number of subjects (n = 720)		684 eyes (684 patients)	36 eyes (36 patients)
Diagnosis	NTG	289 eyes	15 eyes
	PACG	33 eyes	3 eyes
	PEXG	55 eyes	2 eyes
	POAG	93 eyes	6 eyes
	Glaucoma suspects	216 eyes	8 eyes
Mean age (year)		53.75 ± 13.46	53.26 ± 14.69
Female gender (%)		312 (46%)	14 (39%)
Family history of glaucoma (%)		56 (8.0%)	4 (11.1%)
Spherical equivalent (D)		− 1.79 ± 2.90	− 1.65 ± 2.84
CCT (um)		542.06 ± 42.96	544.48 ± 34.80
Baseline IOP (mmHg)		15.46 ± 3.94	16.45 ± 5.66
MD (dB)		− 4.41 ± 5.69	− 5.37 ± 7.54
PSD (dB)		5.32 ± 4.17	4.48 ± 3.75
VFI (%)		88.69 ± 16.61	86.78 ± 22.99
Glaucoma severity	NN	116 eyes	6 eyes
	G1	387 eyes	20 eyes
	G2	112 eyes	6 eyes
	G3	69 eyes	4 eyes

*NTG* normal tension glaucoma; *PACG* primary angle closure glaucoma; *PEXG* pseudoexfoliation glaucoma; *POAG* primary open angle glaucoma; *D* diopters; *CCT* central corneal thickness; *IOP* intraocular pressure; *MD* mean deviation; *PSD* pattern standard deviation; *VFI* visual field index. *NN* unaffected control (MD ≥ 0.0); *G1* mild glaucoma grade (− 6.0 < MD < 0.0); *G2* moderate glaucoma grade (− 12.0 < MD ≤ − 6.0); *G3 *severe glaucoma grade (MD ≤ − 12.0).

**Table 2:** Baseline OCT parameters in glaucoma patients.

Characteristics	Values (mean $\pm$ SD)
BMO-fovea angle$^\circ$	− 6.24 ± 3.54
BMO area ($m{m}^{2}$)	2.35 ± 0.57
BMO-MRW G (um)	215.57 ± 58.37
BMO-MRW T	167.29 ± 48.03
BMO-MRW TS	212.91 ± 74.23
BMO-MRW TI	214.90 ± 74.23
BMO-MRW N	2133.70 ± 67.56
BMO-MRW NS	242.66 ± 73.29
BMO-MRW NI	250.61 ± 82.34
RNFL G	84.85 ± 19.69
RNFL T	69.89 ± 17.35
RNFL TS	113.43 ± 37.43
RNFL TI	111.52 ± 46.85
RNFL N	68.07 ± 18.23
RNFL NS	100.51 ± 30.97
RNFL NI	93.25 ± 28.94

*SD* standard deviation; *OCT* optical coherence tomography; *BMO-MRW* bruch's membrane opening-minimum rim width; *RNFL* retinal nerve fiber layer; *G* Global; *T* temporal; *TS* superotemporal; *TI* inferotemporal; *N* nasal; *NS* superonasal; *NI* inferonasal.

### Workflow of deep learning model for predicting visual field global indexes

We aimed to estimate three VF global indexes, MD, PSD, and VFI among parameters of BMO-MRW and RNFL based on deep learning. The main workflow of our deep learning model for predicting visual field indexes is as follows. First, we extracted numerical parameters of BMO-MRW and RNFL from OCT scan images using Heidelberg licensed software and included the age of patients in the dataset to train and test the deep neural network (DNN) model. A total of 720 eyes from 720 patients were used. Sixteen sub-parameters were used as input parameters in the dataset. Three DNN models were built and trained independently to predict the value of each VF global index: MD, PSD, and VFI. These models had three hidden layers and a single output layer. Exponential linear unit (ELU) was used as activation function. Batch normalization was applied after each hidden layer. The three models were constructed with the same structure. The model for each VF global index (MD, PSD, and VFI) had minor differences in the number of nodes and the degree of regulation in detail. To improve model performance, we applied fivefold cross validation and tuned model hyper-parameters such as learning rate, the degree of regulation, the number of layers, and the number of nodes in each layer. In each fold, the validation set consisted of 137 eyes (137 patients) and the training set consisted of 547 eyes (537 patients). We calculated the MAE in the validation set for each VF global index. To evaluate predicting performance, mean absolute error (MAE), Pearson’s correlation coefficient, and ${R}^{2}$ of each model were calculated, and the results showed in Table 4. The overview of the workflow of each model is illustrated in Fig. 1A. Figure 1B shows the detailed structure of the DNN model.

### Predictive performances of DNN and ML models

**Figure 2:**
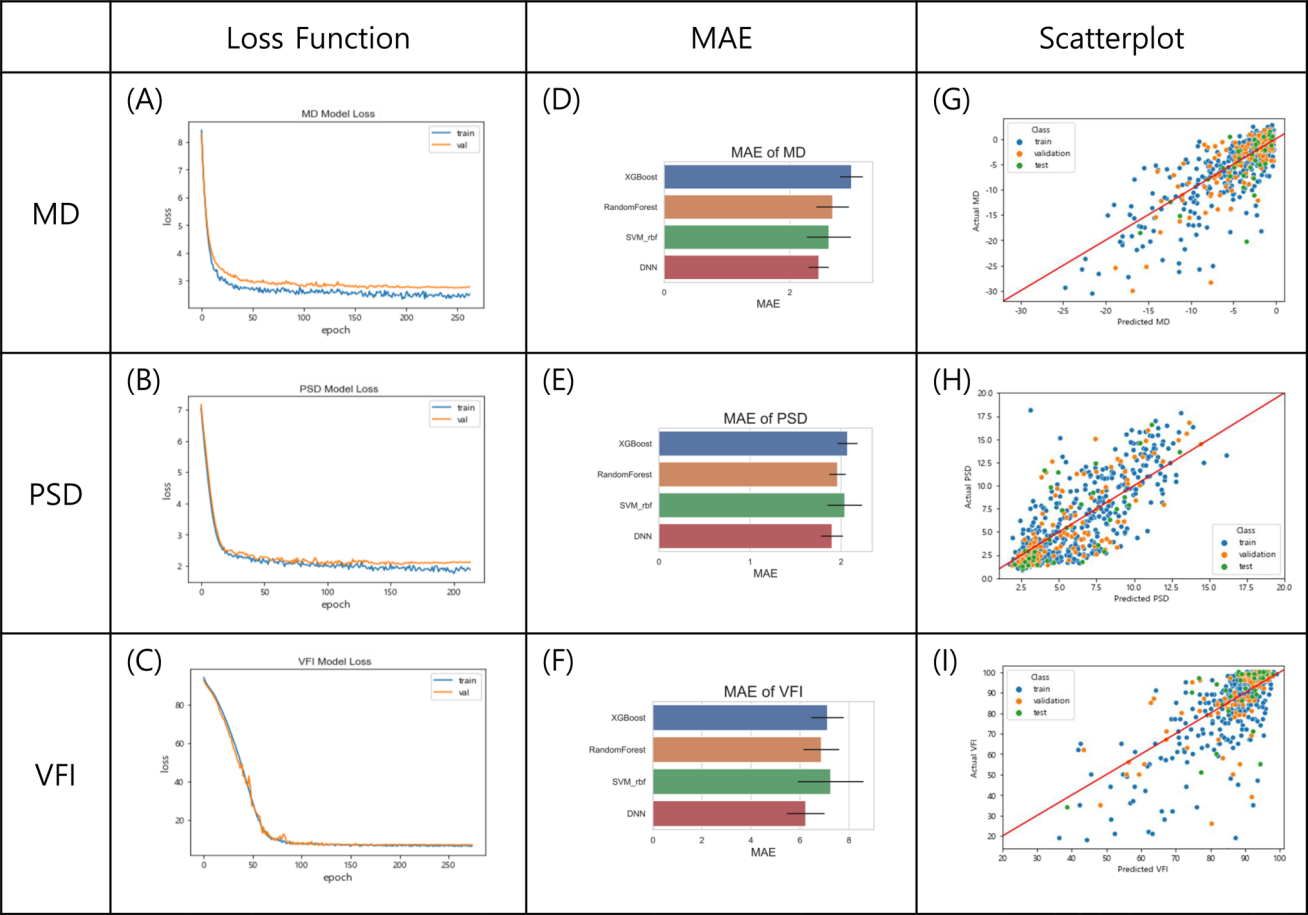
(**A–C**) Loss curve of the DNN model for predicting VF global indexes, MD, PSD, and VFI. The blue line is for the training set and the orange one is for the validation set. The axis x is epoch and the axis y is the value of each loss function. (**D–F**) Comparison of MAE for predicting VF global indexes on fivefold cross validation. In each figure, blue, orange, green, and red bar represent the MAE of XGBoost, Random Forest, SVM with RBF kernel, and the DNN model, respectively. The black bar on all bars means the standard deviation on a fivefold cross validation. The axis $x$ is MAE value. (**G–I**) Scatter plots of deep learning predicted and actual values of three indexes (MD, PSD, and VFI) in the dataset. Blue, orange, and green points mean training set, validation set, and test set, respectively. The axis x means predicted value from the DNN model and the axis y is actual value. VF = visual field; MD = mean deviation; PSD = pattern standard deviation; VFI = visual field index; MAE = mean absolute error; DNN = deep neural network; XGBoost = extreme gradient boosting; SVM = support vector machine; RBF = radial basis function.

To evaluate performance of prediction for our DNN model, we calculated MAE for each VF global index with the validation set. The loss curves of the DNN model for predicting VF global indexes with increasing number of epochs was plotted in Fig. 2A–C. With these loss functions of each index, it was verified that the performance of the DNN model was stable and robust. We also trained other machine learning (ML) models: Random Forest, extreme gradient boosting (XGBoost), and support vector machine (SVM) using Radial Basis Function (RBF) kernel to compare their performances with the DNN model. Results of MAE comparison of DNN and ML models for each VF global index on a fivefold cross validation are demonstrated in Fig. 2D–F. The DNN model showed the lowest MAE VF global indexes. First, the MAE of MD in each model was as follows. The MAE of MD ranged from 1.9 to 2.9 dB for our DNN model, 2.2–2.9 dB for SVM using RBF kernel, 2.3–3.0 dB for Random Forest, and 2.4–3.2 dB for XGBoost. The MAE range of PSD was 1.6–2.0 dB for DNN, 1.8–2.3 dB for XGBoost, 1.8–2.2 dB for Random Forest, and 1.7–2.3 dB SVM using RBF kernel. The MAE of VFI in each model was as follows. The MAE of VFI ranged from 5.0 to 7.0% (6.3–6.9% for Random Forest, 6.5–7.4% for XGBoost, and 6.5–8.0% for SVM using RBF kernel). These results are summarized in Table 3.

**Table 3:** The MAE for DNN model with other machine learning algorithms.

**MD prediction**			
	MAE $\pm$ SD (dB)	Min (dB)	Max (dB)
SVM	$2.64\pm 0.26$	$2.15$	$2.92$
RF	$2.73\pm 0.23$	$2.31$	$3.02$
XGB	$2.97\pm 0.27$	$2.48$	$3.24$
DNN	$2.57\pm 0.33$	$1.95$	$2.87$

**PSD prediction**			
	MAE $\pm$ SD (dB)	Min (dB)	Max (dB)
SVM	$2.04\pm 0.23$	$1.71$	$2.30$
RF	$1.99\pm 0.18$	$1.76$	$2.23$
XGB	$2.08\pm 0.20$	$1.84$	$2.37$
DNN	$1.80\pm 0.14$	$1.63$	$2.00$

VFI prediction			
	MAE$\pm$SD (%)	Min (%)	Max (%)
SVM	$7.09\pm 0.55$	$6.50$	$8.00$
RF	$6.61\pm 0.24$	$6.29$	$6.88$
XGB	$7.01\pm 0.35$	$6.50$	$7.39$
DNN	$6.06\pm 0.66$	$4.95$	$6.97$

*MAE* mean absolute error; *SD* standard deviation; *MD* mean deviation; *PSD* pattern standard deviation; *VFI* visual field index; *SVM* support vector machine; *RF* random forest; *XGB* extreme gradient boosting; *DNN* deep neural network.

### Comparison of actual and DNN predicted values of VF global indexes

Statistical analysis was proceeded to compare actual data of each VF global index with data predicted by the DNN model. Figure 2G–I show scatter plots of predicted and actual values of three indexes (MD, PSD, and VFI) in the dataset. Pearson's correlation coefficient and $${R}^{2}$$ were also measured. Between predicted values and actual values of MD in the fivefold cross validation, Pearson's correlation coefficient was in the range of 0.76 to 0.85 ($$p<0.001)$$. In the PSD estimation, Pearson's correlation coefficient ranged from 0.74 to 0.82 ($$p<0.001)$$. In VFI prediction, the Pearson's correlation coefficient ranged from 0.70 to 0.81 ($$p<0.001)$$. In addition, $${R}^{2}$$ ranges were 0.59–0.65, 0.58–0.66, and 0.58–0.65 for MD, VFI, and PSD, respectively. Statistical results of the DNN on five-fold cross validation are summarized in Table 4.

**Table 4:** Statistical results of the DNN model on five-fold cross validation.

	PCC (min–max)	${R}^{2}$(min–max)
MD	0.76–0.85 ($$p<0.001)$$	0.59–0.65
PSD	0.74–0.82 ($$p<0.001)$$	0.58–0.65
VFI	0.70–0.81 ($$p<0.001)$$	0.58–0.66

*PCC* Pearson’s correlation coefficient; *MD* mean deviation; *PSD* pattern standard deviation; *VFI* visual field index.

### Predictive performances of DNN model according to OCT- derived parameters

We evaluated performances of DNN model for predicting VF index (MD) according to the OCT-based parameters respectively: BMO-MRW alone, RNFL alone, and both BMO-MRW and RNFL combined. The mean absolute error (MAE) of the DNN model based on the parameters of BMO-MRW alone and RNFL alone were 2.72 dB and 2.87 dB, respectively. The performance of the DNN model based on both BMO-MRW and RNFL combined was 2.28 dB of MAE, which showed the smallest value.

### Deep learning predictive performance analysis according to glaucoma severity

**Figure 3:**
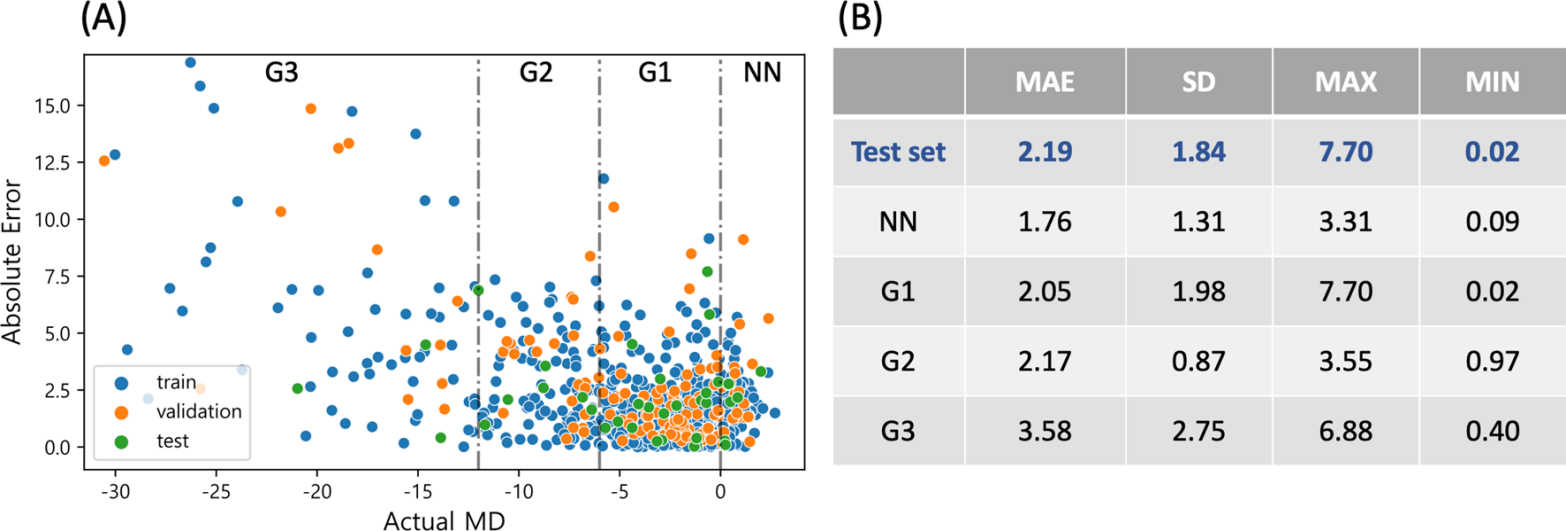
(**A**) Scatter plot of absolute errors of the actual MD and predicted MD for each eye in the data set to evaluate performances of the DNN model according to glaucoma severity. (**B**) The mean absolute error (MAE) of the DNN model according to glaucoma severity in test set. Note that the MAE is the smallest in G1 group, which is early stage of glaucoma. MD = mean deviation; DNN = deep neural network; MAE = mean absolute error; SD = standard deviation; NN = unaffected control (MD $\ge 0.0$); G1 = mild glaucoma grade (− 6.0 $<$ MD $<$ 0.0); G2 = moderate glaucoma grade (− 12.0 $<$ MD $\le$ − 6.0); G3 = severe glaucoma grade (MD $\le$ − 12.0).

To evaluate the predictive performances of the DNN model according to glaucoma severity, we measured absolute errors of the actual value and predicted value of MD for each eye. Figure 3A shows a scatter plot of absolute error showing the prediction performance according to the actual MD values of each eye. The mean absolute error (MAE) of the DNN model was $2.19\pm 1.84$ dB in the test set as shown in Fig. 3B. The prediction performance for each glaucoma severity in the test set is as follows. The MAE for unaffected control (NN; MD ≥ 0.0) was $1.76\pm 1.31$ dB, and the mild glaucoma grade (G1; − 6.0 < MD < 0.0) showed its MAE was $2.05\pm 1.98$ dB. The MAE for moderate glaucoma grade (G2; − 12.0 < MD ≤ − 6.0) class was $2.17\pm 0.87$ dB, and the severe glaucoma grade (G3; MD ≤ − 12.0) was it MAE was $3.58\pm 2.75$ dB. It is noticeable that the MAE of the early stage of glaucoma is the smallest among all the stages of glaucoma.

## Discussion

To our knowledge, the present study was the first to predict all of VF global indexes including MD, PSD, and VFI from OCT-derived parameters of BMO-MRW, a new parameter, and RNFL using a deep learning model. We found that the performance of our DNN model was outstanding along with other machine-learning models in predicting VF global indexes. For all three indexes, the DNN model showed the best performance. We also found that there was a strong correlation between each predicted value and the actual value.

The availability of BMO-MRW obtained from spectral-domain OCT has grown for clinicians. It provides some advantages when compared to the previous standard morphometric optic nerve head analysis confocal scanning laser tomographic measurements^21–23^. Compared to existing ophthalmic examinations, BMO-MRW allows for a more precise geometric assessment of the neuroretinal rim (NRR)^15–17,20^. It has been shown that BMO-MRW is advantageous in providing an accurate reflection of the amount of neural tissue present in the optic nerve^32^. Our previous study reported a high diagnostic performance in discriminating early normal-tension glaucoma (NTG) from glaucoma suspect (GS) (AUC, 0.966) based on a deep learning model using OCT parameters of BMO-MRW, peripapillary RNFL, and color classification of RNFL^25^. Interestingly, BMO-MRW, as a single parameter, provided a higher diagnostic performance (AUC: 0.959) than RNFL alone (AUC: 0.914) and RNFL with its color code classification (AUC: 0.934)^25^. Moreover, BMO-MRW alone showed similar diagnostic performance to that of all three OCT parameters combined. These results suggest that BMO-based optic disc assessment might be a better evaluation for different aspects of the optic disc than conventional disc assessments in the diagnosis of glaucoma. These findings suggest that BMO-MRW is clinically useful in the diagnosis of glaucoma. It might be even better than conventional RNFL. Integrating assessment of BMO-MRW and RNFL is beneficial for better diagnosis of glaucoma based on these findings. However, the integration of these two different parameters is a complex and challenging for human beings, including general physicians other than glaucoma specialists. This is where the latest technology of artificial intelligence can be useful. Recent reports indicate that machine-learning classifiers can aid in clinical practice and efficiently enhance glaucoma diagnosis for general ophthalmologists in the primary eye care setting when there is a lack of glaucoma specialists^33^. The deep learning model can provide rapid diagnostic results in the clinics after inputting ophthalmic examination data without the need for a multi-day analysis. Ultimately, the decision to treat glaucoma is up to the physician, but the deep learning model can suggest a preliminary diagnosis for reference^34^. Moreover, the DNN diagnostic model is more cost-effective clinically easy to access compared to other imaging-based CNN diagnostic programs that require costly equipment, such as workstations with GPUs and take several days to produce results.

A previous study by Park et al.^29^ has predicted VF regional thresholds with deep learning based on inception V3 using combined OCT images of macular ganglion cell-inner plexiform layer (mGCIPL) and peripapillary pRNFL thicknesses maps. They conducted pointwise estimation of VF for a regional analysis. With the deep learning method, the root mean squared error (RMSE) of the entire VF area for all patients was 4.79 $\pm$ 2.56 dB (mean $\pm$ standard deviation). In our study, we estimated global VF. The MAE of MD was found to be 2.57 $\pm$ 0.33 dB. Our results showed lower MAE, suggesting better results in predicting the entire VF threshold. Hemelings et al.^31^ have conducted a study to predict VF MD and 52 threshold values based on a customized CNN model with Xception using peripapillary RNFL map and scanning laser ophthalmoscopy en face images. The MAE for MD estimation the deep learning model was 2.89 dB (range, 2.50–3.30 dB).

In our study, the MAE for MD prediction was 2.57 dB (range, 1.95–2.87 dB). Therefore, the present study showed lower MAE, indicating better results for predicting the entire VF threshold. Christopher et al.^26^ have developed a deep learning system based on ResNet50 to predict MD, PSD, and mean VF sectoral pattern deviation (PD) using image data of RNFL thickness map, RNFL enface image, and confocal scanning laser ophthalmoscopy image. In MD estimation, the deep learning model with RNFL enface image achieved the highest performance with ${R}^{2}$ of 0.70 (range, 0.64–0.74) and MAE of 2.5 dB (range, 2.3–2.7 dB). In PSD estimation, ${R}^{2}$ was 0.61(range, 0.55–0.66) and MAE was 1.5 dB (range, 1.4–1.6 dB). Our deep learning model, which utilized combined parameters of RNFL and BMO-MRW, demonstrated similar performance to other previous studies. It could also predict additional VF global indexes such as VFI. Results of our study were highly comparable to those of previous research, thus having a significant meaning. Yu et al. have used 3D CNN model to estimate VF global indexes of MD and VFI, but not all three indexes from combining macula and optic disc OCT scans in healthy, glaucoma suspect, and glaucoma patients^27^. Each global index of VF test has its own advantage, and thus, only one index cannot tell all the aspects of the entire VF results. For example, MD is useful to estimate the overall stage of glaucoma. On the other hand, PSD reflects the focal VF defect in an early stage of glaucoma, which is beneficial in the diagnosis of early glaucoma.

Using the deep learning model based on macular and optic nerve head scans, the MAE was 1.57 dB for MD and 2.7% for VFI. Yu et al. have shown great results with a larger number of images. However, their study included multiple visit data from one patient to have a larger number of images. We used single visit data from each subject, which might be more independent and reliable. Moreover, we used data extracted from OCT using lighter and cost-effective model to predict VF global indexes. Our results were quite comparable to results of the study by Yu et al. using images from OCT with a more complicated model. Results of VFI seemed to be better in the study by Yu et al. (2.7 dB for VFI). However, considering VFI percentage in our study, results were substantially good. The VFI reflects RGC loss and function as a percentage, with central points having more weights^35^. It is expressed as a percentage of remaining proportion of visual function. It is a reliable index on which glaucomatous visual field severity staging can be based. VFI can also be used to calculate the rate of progression which is shown in trend-based glaucoma progression analysis of Humphrey Field Analyzer software^36^. While VFI is important in the management of glaucoma, previous studies have predicted that this global index (VFI) is rare to be found in the field of AI (artificial intelligence) using deep learning methods. Most of previous studies have mainly focused on predicting MD as a global index from different images of OCT or HRT device^26–31^. Our study also had a significant meaning in that we predicted VFI as a global index from extracted OCT data. This has not been reported before in the field of AI using deep learning method.

The result of the current study has a significant clinical meaning in that it provides summary outline numbers of functional VF test from structural OCT test. OCT test is objective. It offers quantitative values of optic nerve head parameters. However, VF requires patient cooperation, a relatively long time, and designated space to be performed. Sometimes and quite frequently, VF test results are not available at the time of clinical practice. Since VF test also requires cognitive ability and motor reaction, for old patients and those with dementia or stroke and/or those with motor disability, VF test cannot be performed correctly. Moreover, in some clinics, VF tests need appointment. They cannot be done at the first visit because all appointed VF tests are being performed at that time. If that patient cannot come back in a short time, VF test can be delayed for a very long time. Thus, correct diagnosis of glaucoma or decision for the disease progression is difficult to be made. In such situations, if summary results of VF test could be predicted from OCT test without actually performing the VF test, it could be clinically very helpful in the management of glaucoma. Especially, in our deep learning VF global indexes prediction model, the performance of the prediction was the best in early stage of glaucoma based on the MAE as shown in Fig. 3A. Early stage of glaucoma or glaucoma suspects usually visit glaucoma clinic to be diagnosed of glaucoma for the first time and in these cases VF test results are necessary. Our relatively quick DNN model may be also useful in these situations, which frequently occur in clinics.

NTG comprises the majority (76.3%) among patients with POAG in Asian populations as reported by previous population-based studies^37^. Thus, information regarding NTG is clinically important for Asians. It applies to Asian countries and also other countries elsewhere with a substantial proportion of Asian population. However, previous deep-learning studies rarely included NTG. It is difficult to find studies including data of NTG or those even classified NTG. As previous deep-learning studies including data of NTG are scarce, the current study might have a significant meaning to be added in the literature for providing additive information and future deep-learning studies in the field of glaucoma.

The current study had several limitations. First of all, there are potential limitations owing to its retrospective design. We included only those who had taken both RNFL and BMO-MRW tests with an acceptable images quality. In addition, only those who had reliable VF tests were included. The impact of the subject selection on our results remains unclear. Second, the study was conducted at a referral university hospital within the province using a hospital-based design, rather than a population-based approach.

The individuals included in the study may not be fully representative sample of the general population. Additionally, this study included only Korean patients. Thus, results of our study, including NTG, might not be applicable to other ethnic groups. Third, it should be considered that the sample size of this study is relatively small. Although 720 subjects with either glaucoma or GS were included in this study, this number might not be insufficient to train or test the performance to predict a single test result from single device data. Other studies with large number used both eyes from multiple visits. However, we used only one randomly selected eye from one person from a single visit. Our data might be more independent and more reliable/correct than previous studies. If we have included both eyes from multiple visits, the number of data could be much larger, for example, six times. Finally, the analysis of OCT images utilizing deep neural network (DNN) in this study was based on the extraction of numerical data from the images rather than using direct images. However, it is still meaningful in that clinicians can use deep-learning models with free open-sources to obtain prompt results and get aid in the management of glaucoma. This approach is more economically feasible than using convolutional neural networks (ConvNets) for image analysis, which can be costly to achieve high accuracy. We might consider developing our own program to be used in clinical practice to aid preliminary diagnosis from direct OCT-image analysis employing ConvNets in future studies achieving accurate performance.

In conclusion, our DNN model showed high performance in predicting VF global indexes of MD, PSD, and VFI based on OCT-derived parameters of BMO-MRW, a new parameter, and RNFL. Prediction based on VFI was the highest, followed by that based on MD and PSD using our DNN model in GS and glaucoma patients. Our DNN model might be beneficial in clinical practice in the management of glaucoma including diagnosis and monitoring progression. Given that our DNN model provides prompt outputs, it has the potential to the particularly valuable in settings where there are no glaucoma specialists available, such as primary eye care. Nonetheless, a more conclusive determination would require a larger, multi-center study with a substantial patient cohort.

## Material & methods

### Ethics statement

This retrospective observational, cross-sectional study was conducted in accordance with the tenets of the Declaration of Helsinki. It was approved by the Institutional Review Board (IRB) of Gyeongsang National University Changwon Hospital, Gyeongsang National University School of Medicine. The requirement for informed consent was waived by the IRB of Gyeongsang National University Changwon Hospital due to its retrospective nature.

### Subjects

Among 1487 patients with glaucoma and glaucoma suspects who were evaluated between February 2016 and December 2021 in a glaucoma clinic at Gyeongsang National University Changwon Hospital, a total of 720 eyes (720 subjects) were included. Glaucoma diagnosis included early NTG, PACG, PEXG, POAG, and GS. Subjects consisted of 224 eyes of those with GS, 245 eyes of those with early NTG, 59 eyes of those with moderate stage of NTG, 36 eyes of those with PACG, 57 eyes of those with PEXG, and 99 eyes of those with POAG. The study included only those participants who met the diagnostic criteria below and demonstrated reliable results for both BMO-MRW and RNFL.

Diagnosis of glaucoma was assessed by a single glaucoma specialist (H-k Cho) applying consistent criteria. To diagnose NTG, patients needed to meet specific criteria, including having an IOP ≤ 21 mmHg without treatment who demonstrated glaucomatous optic disc injury and corresponding VF loss, an open-angle assessed by gonioscopic inspection, and no other underlying cause of optic disc injury other than glaucoma^38^. Early NTG was defined as the VF test results of MD > − 6.0 dB. PACG was determined as eyes with shallow anterior chamber (appositional contact between the peripheral iris and the trabecular meshwork (TM) > 270 degrees on gonioscopy and showed glaucomatous optic disc damage (decline of NRR with a vertical cup-to-disc ratio of 0.7 or an asymmetry between eyes of 0.2, or notching ascribe to glaucoma) and showing corresponding visual field defects^39^. To diagnose PEX glaucoma, the criteria included the observation of PEX material at the margin of the pupil and on the anterior lens capsule after maximal pupil dilatation, along with the presence of baseline IOP of at least 22 mmHg, glaucomatous optic nerve head damage, visual field loss consistent with optic disc injury, and the absence of other conditions causing secondary glaucoma^40^. POAG was defined as a patient with a baseline IOP of more than 21 mmHg prior to treatment who showed findings of glaucomatous optic nerve head injury and corresponding VF loss, an open-angle assessed by gonioscopic inspection, and no other underlying cause for optic nerve head injury besides glaucoma^1^.

The exclusion criteria were as follows: low-quality image scans resulting from eyelid blinking or poor fixation, history of optic neuropathies aside from glaucoma or an acute angle-closure crisis that could affect the thickness of the RNFL or BMO-MRW (e.g., optic neuritis, acute ischemic optic neuritis), history of any intraocular surgery except for uneventful phacoemulsification, and retinal disease associated with retinal swelling or edema and subsequent RNFL or BMO-MRW swelling. Preperimetric glaucoma was excluded from the current study. Subjects were not excluded by axial length or refractive error, or the size of optic disc for the present study.

### Optical coherence tomography

Imaging of spectral-domain OCT was accomplished using the Glaucoma Module Premium Edition. Radial B-scans of 24 in number were acquired to analyze BMO-MRW. Among three scan circle diameters (3.5, 4.1, and 4.7 mm), a scan circle diameter of 3.5 mm was chosen for peripapillary RNFL thickness measurement. Only those images that were correctly centered and accurately segmented and quality scores ≥ 20 were selected for this study. Images taken with OCT were aligned in FoBMO axis, that is an individual specific axis that measures between the center of BMO and the fovea of macula. Employing this FoBMO axis could enable more correct analysis of Garway-Heath sector considering cyclotorsion of each individual and more precise analysis compared with normative database than the existing way of using only simple clock-hour locations.

### Perimetry

We used a Humphrey Field Analyzer (HFA model 840; Humphrey Instruments Inc.) for perimetry with a central 30-2 program of Swedish Interactive Threshold Algorithm standard strategy. A reliable VF test had to qualify the following criteria: false-positive rate < 15%; false-negative rate < 15%; and fixation loss less than 20%.

### Data preprocessing

The dataset consisted of OCT parameters and age of 720 eyes. Parameters included the following: age, BMO Area, BMO-MRW Global, BMO-MRW Temporal, BMO-MRW superotemporal (TS), BMO-MRW inferotemporal (TI), BMO-MRW Nasal, BMO-MRW superonasal (NS), BMO-MRW inferonasal (NI), RNFL Mean Global, RNFL Mean Temporal, RNFL Mean TS, RNFL Mean TI, RNFL Mean Nasal, RNFL Mean NS, and RNFL Mean NI. Each feature was standardized by its mean and standard deviation to make learning process more efficiently. Stratified sampling was used to compensate for the relatively small size of dataset to be divided randomly. Out of 720 eyes, 684 eyes were used to construct a train set (95%) and 36 eyes were used to form a test set (5%). Since test data were used for comparing prediction performances of each model, they contained five percent of the dataset. K-fold cross validation (k = 5) was applied. The train set was re-slitted to a ratio of 8:2 for train set (n = 541) and validation set (n = 137). Programming language Python version 3.9.7 (https://www.python.org/) and the package Scikit-learn 0.24.2 (https://scikit-learn.org/) were used to preprocess all data.

### Machine learning algorithm

Machine learning means the use of an algorithm to make prediction not based on logics but based on data. Models rarely had any explicit rule or strict logic. Instead, they generate results by using the data^41^. The process of getting results can vary depending on the method of the ML algorithm. In our study, several ML models, Random Forest, XGBoost, SVM, and SVM with Radial Basis Function, were used and compared with a DNN model. Random Forest algorithm is one of the mainly used ML algorithms for tasks of classification and regression. It combines several decision trees and makes predictions by using voting system which averages all decision trees’ results^42^. XGBoost is also based on decision tree like Random Forest. However, it implements a boosting process which is the ensemble learning technique of building several models sequentially^43^. SVM is an ML algorithm that maps data from the feature space into the kernel space^44^. We also used SVM with RBF kernel^45^.

### Deep neural network architecture

A DNN is an artificial neural network with more than two hidden layers and a non-linear activation function. DNN proceeds learning process by repeating feedforward and backpropagation^46^. We built our model using open-source neural network APIs, Keras (https://keras.io/), and TensorFlow (https://www.tensorflow.org/). Each model was built slightly differently because each VF global index had different meaning, values, and distributions. According to the index, we made three DNN models in this study: MD prediction model (MD model), PSD prediction model (PSD model), and VFI prediction model (VFI model). These models had the same number of layers: a single input layer, three hidden layers, and an output layer as shown in Fig. 1B. Each model received input data with 16 parameters which consisted of age and other ocular parameters extracted from OCT scans and related to BMO-MRW and RNFL. Batch Normalization was used after each hidden layer^47^. An ELU function was used as an activation function^48^. To prevent overfitting, l2-regularizer was used. An adaptive moment estimation optimizer (Adam) (learning rate = 0.05) was used for each model^49^. Learning rate decay method was applied. MSE was used for its loss function. Architectures of these models used in this study are shown in Fig. 1.

### Statistical analysis

To evaluate the performance of the deep-learning model, MAE was utilized. MAE was evaluated to determine the performance of a regression model interpretably. It is generally known as more intuitive and easier to interpret than root mean squared error. MAE is the average of the absolute value of the deviation. The formula to calculate MAE for each indicator is shown as follows:

$$MAE= \frac{1}{n}\sum_{i=1}^{n}\left|\text{Actual value}-\text{Predicted value}\right|$$

We also calculated Pearson's correlation coefficient ($\rho$) and ${R}^{2}$ to evaluate how our models were trained and whether they showed convincing prediction^50^. All statistical analyses were performed using programming language Python version 3.9.7 (https://www.python.org/) and the package Scikit-learn 0.24.2 (https://scikit-learn.org/).

## Data Availability

Dataset used in this study might be obtained from Hyun-kyung Cho (MD, PhD) upon reasonable request.
